# Microwave-assisted simple synthesis of 2-anilinopyrimidines by the reaction of 2-chloro-4,6-dimethylpyrimidine with aniline derivatives†

**DOI:** 10.1039/d0ra00833h

**Published:** 2020-03-25

**Authors:** Cristina Campestre, György Keglevich, János Kóti, Luca Scotti, Carla Gasbarri, Guido Angelini

**Affiliations:** Department of Pharmacy, University “G. d’Annunzio” of Chieti-Pescara via dei Vestini 66100 Chieti Italy guido.angelini@unich.it +39-0871-3554785; Department of Organic Chemistry and Technology, Budapest University of Technology and Economics 1521 Budapest Hungary; Spectroscopic Research Division, Gedeon Richter Plc. 1475 Budapest Hungary; Department of Medical, Oral and Biotechnological Sciences, University “G. d’Annunzio” of Chieti-Pescara via dei Vestini 66100 Chieti Italy

## Abstract

A series of 2-anilinopyrimidines including novel derivatives has been obtained from 2-chloro-4,6-dimethylpyrimidine by aromatic nucleophilic substitution with differently substituted anilines under microwave conditions. The substituents had a significant impact on the course and efficiency of the reaction. The results reported herein demonstrate the efficacy of microwaves in the synthesis of the title heterocyclic compounds as compared to the results obtained with conventional heating. The 2-anilinopyrimidines described are of potential bioactivity.

## Abstract 2

A rapid, one pot, green approach by microwave heating in ethanol provides a new method to obtain active and new potentially active compounds in high yields.

## Graphical abstract

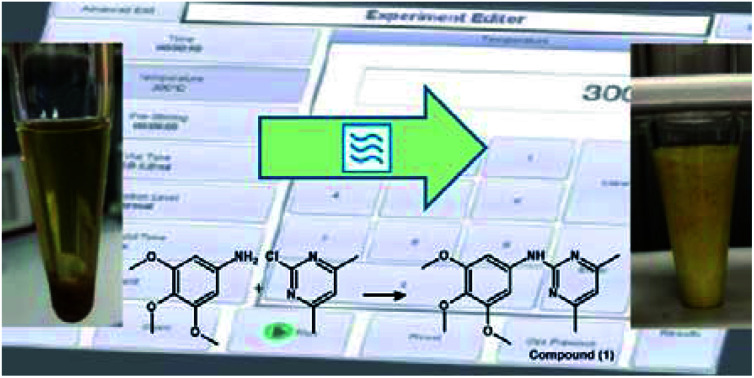

## Introduction

The biological activity of anilinopyrimidines as fungicides and pesticides is well-known and widely reported.^1–7^ Recently, a few 2-anilinopyrimidine derivatives have been evaluated as kinase inhibitors having antiproliferative activity against cancer cell lines.^8–11^ The role of this class of compounds in the generation of supramolecular networks for molecular recognition has also been demonstrated.^12^

Different methods have been proposed to synthesize anilinopyrimidines: (a) the cyclization between guanidines (generally obtained from isothiourea salts and alkyl(aryl)amines in the presence of strong bases) and β-diketones, ethyl acetoacetate or ethyl cyanoacetate under refluxing for several hours (b) the transition metal-free cross-coupling reactions, (c) the aromatic nucleophilic substitution of halogen pyrimidines or substituted heterocycles having an alkylsulfonyl group with anilines.^13–16^ In particular, the substitution of the halogen atom in 2-aminopyrimidines by alkyl- or arylamines occurs under acidic conditions.^17,18^ The main disadvantage of these procedures is the requirement for drastic conditions and long reaction times.

In the last years a large number of heterocyclic compounds including anilinopyrimidines has been synthesized by microwave (MW) irradiation.^19–21^ The main advantage of the use of this technique is the decrease of the reaction time from several hours to a few minutes or seconds in comparison to the results obtained on conventional heating.^22,23^ Moreover, less by-products are formed in MW-assisted reactions.^24^ However, in most cases, it is not possible to predict if a given reaction will be improved under MWs or not.^25^ At the same time, if the energetics of the target reaction is known, it is possible to judge in advance about the appropriateness of the application of MWs. The ideal subject of MW-promoted reactions are those that have a relatively high enthalpy of activation, and are thermoneutral.^26–28^

In this article we describe the simple synthesis of a series of anilinopyrimidine derivatives including three new compounds by the reaction of 2-chloro-4,6-dimethylpyrimidine with substituted anilines under MW irradiation. This method represents a novel approach to the synthesis of the target compounds, and allows high yields in eco-friendly conditions. Recently, some green and recyclable reaction media have been proposed, such as water, PEG-200, and 2-methyltetrahydrofuran.^29–31^ The use of ethanol, one of the environmentally preferable solvents, confers the eco-friendly character to the synthesis of the investigated compounds.^32–34^

## Results and discussion

**Scheme 1:**
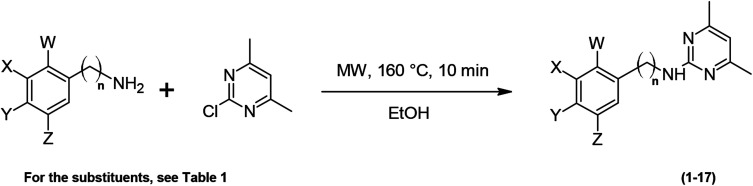
MW-assisted synthesis of the compounds (1–17).

Recently, it has been demonstrated that acid catalysis promoted the reaction of chloro-*N*-heterocycles with anilines.^19,35^ The reaction between 2-chloro-4,6-dimethylpyrimidine and substituted anilines in ethanol at 160 °C under MW irradiation for 10 min, is an aromatic nucleophilic substitution taking place *via* the corresponding Meisenheimer complexes, and affording 2-anilinopyrimidines. An aminopyrimidine from benzylamine has also been synthesized as shown in Scheme 1.

Yields of the 2-anilinopyrimidine derivatives (1–16) and benzylaminopyrimidine (17) are listed in Table 1. All the target compounds were prepared with high yields (71–99%) except compound (10). Pure products were obtained after column chromatography, and their structure was confirmed by ^1^H and ^13^C-NMR as well as mass spectral data.

**Table 1:** Preparative data and melting points for the synthesized products (1–17)

Compound	X	Y	Z	W	*n*	Yield/%	mp (lit)/°C
1	OCH_3_	OCH_3_	OCH_3_	H	0	90	167.5–169
2	H	H	H	H	0	91	96–98 (96–97)^21^
3	H	OCH_3_	H	H	0	90	90–92 (88–89)^21^
4	H	OH	H	H	0	92	170–172 (170–172)^36^
5	H	CH_3_	H	H	0	99	116–117 (107–108)^36^
6	H	Br	H	H	0	98	123–124 (123–124)^36^
7	H	Cl	H	H	0	82	118–120 (153)^36^
8	H	F	H	H	0	71	91–93 (91–93)^36^
9	H	CF_3_	H	H	0	87	106.5–108
10	H	NO_2_	H	H	0	39	221–223 (230)^36^
11	H	C_6_H_5_	H	H	0	91	114–115.5
12	H	N=N–C_6_H_5_	H	H	0	91	159.5–161
13	CH_2_OH	H	H	H	0	97	106–108
14	F	H	H	H	0	97	132–134 (132–134)^36^
15	OH	H	H	H	0	85	137.5–139 (157)^36^
16	H	H	H	CH_3_	0	90	111–112 (92–93)^36^
17	H	H	H	H	1	95	109–111 (111–112)^21^

**Scheme 2:**
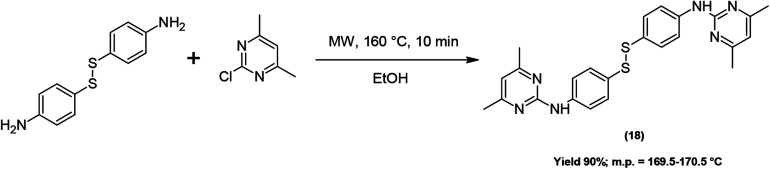
MW-assisted synthesis of bis-derivative (18).

The bis-derivative (18) was obtained according to Scheme 2, under the same conditions as compounds (1–17), starting from 2-chloro-4,6-dimethylpyrimidine and 4,4′-dithiobis(benzenamine).

In order to verify the role of MWs in the investigated reactions, compound (1) was also synthesized on conventional heating at reflux for 72 h. After the work-up described in the general procedure, product (1) was isolated in a yield of 68% that is significantly lower than that obtained by MW irradiation (90%). It has been demonstrated that in the MW-assisted synthesis, the polarity and polarizability of the reagents, transition states and intermediates may influence the absorption of MWs and hence the heating.^37^

The presence of substituents, their electronic effects and their position in the aromatic ring may influence the properties of the compounds, and the rate of their reactions.^38–43^ In the investigated S_N_Ar reactions, the electron-donating effect (+I) of the alkyl group increases the nucleophilic nature of the amino group of the aniline moiety, as it was demonstrated by the high yields obtained for compounds (5) and (16) due to the presence of the CH_3_ group in the *para* or *ortho* position, respectively (Table 1). A similar effect was observed in the phenyl substituted instance (11). In case of products (3) and (4), the high yields of 90 and 92%, respectively, are the consequence of the electron-releasing MeO and HO groups. These substituents can promote the reactivity of the aniline molecule because of the major +M effect *versus* the −I effect. On the other hand, regarding compound (10), in which the nitro group posseses an −M effect, nucleophilicity of the amino group is decreased, as it was demonstrated by the lower yield of 39%.

Compounds (1), (2) and (3) are well-known molecules: heterocycle (1) has been recently patented for its antimitotic activity as a topical formulation against psoriasis,^44^ while derivatives (2) and (3) are commercial fungicides pyrimethanil and andoprim, respectively.^2,45,46^ In this work, these species and their analogues have been synthesized in a one pot reaction instead of the guanidine route.^14^ To the best of our knowledge, compounds (11), (12) and (18) are reported for the first time.

## Conclusions

MW irradiation allowed rapid and high yield “green” synthesis of a series of 2-anilinopyrimidines. In this work, 18 products were prepared, including new compounds (11), (12) and (18) and the benzylamine derivative (17). The electronic effect of the substituents had an impact on the outcome of the reaction by influencing the nucleophilic character of the amino group of the aniline molecule. As N-heterocycle-based molecules are important due to their applicability in medicinal chemistry,^47^ the compounds synthesized beyond the antimitotic (1) and fungicidal (2) and (3) may also posses bioactive properties.

## Experimental

### Material and methods

All the reagents, were purchased from commercial suppliers and employed without further purification. Reactions were monitored by thin layer chromatography (TLC) on silica gel plates (60 F254) that were visualized under UV light (254 nm). The silica gel (Kieselgel 4°, 0.04–0.063 mm) used in the column chromatography was purchased from Merck. The MW-assisted reactions were performed using a Biotage Initiator Plus oven. Infrared absorption spectra were recorded on a Varian 1000 Fourier-transform infrared spectrometer. The ^1^H and ^13^C-NMR spectra were recorded on a Varian-Mercury 300 spectrometer, operating at a frequency of 300 MHz and 75 MHz, respectively. Melting points were determined on a Büchi B-540 apparatus and are uncorrected. Electrospray high-resolution MS measurements were performed on a Thermo Velos Pro Orbitrap Elite Hybrid Mass spectrometer (Thermo Fisher Scientific, Bremen, Germany). The ionization method was ESI and operated in positive ion mode. The capillary temperature was set at 275 °C. Samples were infused into the ESI source MeOH solutions at a flow rate of 3 μL min^−1^. Resolving power of 60 000 (FWHM) at *m*/*z* 400. Data acquisition and analysis were accomplished with Xcalibur software version 3.0 (Thermo Fisher Scientific Inc.).

### General procedure for the synthesis of compounds (1–17)

The appropriate aniline (1.0 mmol), 2-chloro-4,6-dimethylpyrimidine (114 mg, 0.80 mmol), and ethanol (4 mL) were placed in a 5 mL reaction vial, sealed and irradiated at 160 °C for 10 min under magnetic stirring. The solid residue was taken up in 30 mL of CH_2_Cl_2_, and the solution washed with 0.25 M Na_2_CO_3_ (2 × 20 mL) and dried over Na_2_SO_4_. The solvent was evaporated, and the crude product so obtained purified by silica gel column chromatography. The yields were calculated combining the fractions having only one spot on TLC. According to the NMR spectra, purity of the compounds is ≥ 99%. The following products were thus prepared:

#### 4,6-Dimethyl-*N*-(3,4,5-trimethoxyphenyl)-2-pyrimidinamine (1)

Yield = 90%; white solid; mp 167.5–169 °C. It was obtained from 183 mg of 3,4,5-trimethoxyaniline and purified by column chromatography starting from hexane to hexane–ethyl acetate (8 : 2) as the eluent.

IR (KBr) *ν*_max_ cm^−1^ 3317, 2951, 1612, 1593, 1544; ^1^H-NMR (CDCl_3_): *δ* 2.37 (s, 6H, 2CH_3_), 3.80 (s, 3H, OCH_3_), 3.85 (s, 6H, 2 OCH_3_), 6.49 (s, 1H, Ar Pyr), 6.99 (s, 2H, Ar), 7.32 (s, 1H, NH); ^13^C-NMR (CDCl_3_): *δ* 23.9, 56.0, 61.1, 96.8, 111.6, 135.9, 153.3, 159.3, 167.6; ESI-HRMS [M + H]^+^ found = 290.14950, C_15_H_20_N_3_O_3_ requires = 290.14992.

#### 4,6-Dimethyl-*N*-phenyl-2-pyrimidinamine (2)

Yield = 91%; yellow solid; mp 96–98 °C. It was obtained from 93 mg of aniline and purified by column chromatography starting from hexane to hexane–ethyl acetate (8 : 2) as the eluent.

IR (KBr) *ν*_max_ cm^−1^ 3260, 2967, 1614, 1589, 1566, 1548; ^1^H-NMR (CDCl_3_): *δ* 2.37 (s, 6H, 2CH_3_), 6.48 (s, 1H, Ar Pyr), 6.98–7.03 (m, 1H, Ar), 7.29–7.34 (m, 2H, Ar), 7.43 (s, 1H, NH), 7.66–7.69 (m, 2H, Ar); ^13^C-NMR (CDCl_3_): *δ* 23.9, 111.7, 118.9, 122.1, 128.9, 139.9, 159.7, 167.6; ESI-HRMS [M + H]^+^ found = 200.11781, C_12_H_14_N_3_ requires = 200.11822.

#### *N*-(4-Methoxylphenyl)-4,6-dimethyl-2-pyrimidinamine (3)

Yield = 90%; brown solid; mp 90–92 °C. It was obtained from 123 mg of 4-anisidine and purified by column chromatography starting from hexane to hexane–ethyl acetate (8 : 2) as the eluent.

IR (KBr) *ν*_max_ cm^−1^ 3266, 2960, 1602, 1583, 1563, 1545; ^1^H-NMR (CDCl_3_): *δ* 2.33 (s, 6H, 2CH_3_), 3.78 (s, 3H, OCH_3_), 6.43 (s, 1H, Ar Pyr), 6.86 (d, *J* = 8.7, 2H, Ar), 7.34 (s, 1H, NH), 7.53 (d, *J* = 8.7, 2H, Ar); ^13^C-NMR (CDCl_3_): *δ* 23.9, 55.6, 111.2, 114.2, 121.1, 133.1, 155.2, 159.9, 167.6; ESI-HRMS [M + H]^+^ found = 230.12824, C_13_H_16_N_3_O requires = 230.12879.

#### 4-[(4,6-Dimethylpyrimidine-2-yl)-amino]-phenol (4)

Yield = 92%; brown solid; mp 170–172 °C. It was obtained from 109 mg of 4-aminophenol and purified by column chromatography starting from hexane to and hexane–ethyl acetate (6 : 4) as the eluent.

IR (KBr) *ν*_max_ cm^−1^ 3217, 2958, 1598, 1585, 1561, 1546; ^1^H-NMR (CD_3_OD): *δ* 2.27 (s, 6H, 2CH_3_), 4.88 (s, 1H, NH) (s, 1H, OH), 6.47 (s, 1H, Ar Pyr), 6.73 (d, *J* = 8.9, 2H, Ar), 7.39 (d, *J* = 8.9, 2H, Ar), ^13^C-NMR (CD_3_OD): *δ* 26.3, 114.1, 118.7, 125.8, 135.9, 156.6, 164.3, 171.65; ESI-HRMS [M + H]^+^ found = 216.11271, C_12_H_14_N_3_O requires = 216.11314.

#### 4,6-Dimethyl-*N*-(4-methylphenyl)-2-pyrimidinamine (5)

Yield = 99%; brown solid; mp 116–117 °C. It was obtained from 107 mg of 4-toluidine and purified by column chromatography starting from hexane to hexane–ethyl acetate (9 : 1) as the eluent.

IR (KBr) *ν*_max_ cm^−1^ 3263, 2964, 1609, 1585, 1565, 1541; ^1^H-NMR (CDCl_3_): *δ* 2.31 (s, 3H, CH_3_), 2.35 (s, 6H, 2CH_3_), 6.45 (s, 1H, Ar Pyr), 7.12 (d, *J* = 8.1, 2H, Ar), 7.43 (s, 1H, NH), 7.54 (d, *J* = 8.1, 2H, Ar); ^13^C-NMR (CDCl_3_): *δ* 20.8, 23.9, 111.3, 119.2, 129.4, 131.6, 137.3, 159.8, 167.6; ESI-HRMS [M + H]^+^ found = 214.13349, C_13_H_16_N_3_ requires = 214.13387.

#### *N*-(4-Bromophenyl)-4,6-dimethyl-2-pyrimidinamine (6)

Yield = 98%; white solid; mp 123–124 °C. It was obtained from 172 mg of 4-bromoaniline and purified by column chromatography starting from CH_2_Cl_2_ to CH_2_Cl_2_–isopropanol (95 : 5) as the eluent.

IR (KBr) *ν*_max_ cm^−1^ 3408, 2962, 1595, 1564, 1519; ^1^H-NMR (CDCl_3_): *δ* 2.33 (s, 6H, 2CH_3_), 6.47 (s, 1H, Ar Pyr), 7.36 (d, *J* = 8.7, 2H, Ar), 7.54 (d, *J* = 8.7, 2H, Ar), 7.67 (s, 1H, NH); ^13^C-NMR (CDCl_3_): *δ* 23.9, 111.9, 114.1, 120.4, 131.6, 139.1, 159.4, 167.6; ESI-HRMS [M + H]^+^ found = 278.02861, C_12_H_13_BrN_3_ requires = 278.02874.

#### *N*-(4-Chlorophenyl)-4,6-dimethyl-2-pyrimidinamine (7)

Yield = 82%; brown solid; mp 118–120 °C. It was obtained from 128 mg of 4-chloroaniline and purified by column chromatography starting from CH_2_Cl_2_ to CH_2_Cl_2_–isopropanol (98 : 2) as the eluent.

IR (KBr) *ν*_max_ cm^−1^ 3317, 2949, 1612, 1593, 1544,; ^1^H-NMR (acetone d6): *δ* 2.31 (s, 6H, 2CH_3_), 6.59 (s, 1H, Ar Pyr), 7.29 (d, *J* = 8.8, 2H, Ar), 7.95 (d, *J* = 8.8, 2H, Ar), 8.59 (s, 1H, NH); ^13^C-NMR (acetone d6): *δ* 23.9, 112.2, 120.9, 126.1, 129.1, 140.9, 160.7, 168.2; ESI-HRMS [M + H]^+^ found = 234.07884, C_12_H_13_ClN_3_ requires = 234.07925.

#### *N*-(4-Fluorophenyl)-4,6-dimethyl-2-pyrimidinamine (8)

Yield = 71%; brown solid; mp 91–93 °C. It was obtained from 111 mg of 4-fluoroaniline and purified by column chromatography starting from CH_2_Cl_2_ to CH_2_Cl_2_–isopropanol (98 : 2) as the eluent.

IR (KBr) *ν*_max_ cm^−1^ 3267, 2964, 1614, 1595, 1566, 1550; ^1^H-NMR (CDCl_3_): *δ* 2.34 (s, 6H, 2CH_3_), 6.47 (s, 1H, Ar Pyr), 6.95–7.01 (m, 2H, Ar), 7.48 (s, 1H, NH), 7.56–7.60 (m, 2H, Ar); ^13^C-NMR (CDCl_3_): *δ* 23.8, 111.6, 115.3 (d, *J* = 22), 120.6 (d, *J* = 6.7), 135.8, 158.3 (d, *J* = 242), 159.4, 167.6; ESI-HRMS [M + H]^+^ found = 218.10849, C_12_H_13_FN_3_ requires = 218.10880.

#### 4,6-Dimethyl-*N*-[4-(trifluoromethyl)phenyl]-2-pyrimidinamine (9)

Yield = 87%; yellow solid; mp 106.5–108 °C. It was obtained from 161 mg of 4-trifluoromethylaniline and purified by column chromatography using hexane–ethyl acetate (9 : 1) as the eluent.

IR (KBr) *ν*_max_ cm-1 3435, 2989, 1618, 1600, 1598, 1535; ^1^H-NMR (CDCl3): *δ* 2.39 (s, 6H, 2CH_3_), 6.55 (s, 1H, Ar Pyr), 7.53 (d, *J* = 8.4, 2H, Ar), 7.73 (s, 1H, NH), 7.78 (d, *J* = 8.4, 2H, Ar); ^13^C-NMR (CDCl_3_): *δ* 23.9, 112.5, 118.1, 122.5, 126.1, 143.1, 159.2, 167.8; ESI-HRMS [M + H]^+^ found = 268.10489, C_13_H_13_F_3_N_3_ requires = 268.10561.

#### 4,6-Dimethyl-*N*-(4-nitrophenyl)-2-pyrimidinamine (10)

Yield = 39%; yellow solid; mp 221–223 °C. It was obtained from 138 mg of 4-nitroaniline and purified by column chromatography starting from hexane to hexane–ethyl acetate (8 : 2) as the eluent.

IR (KBr) *ν*_max_ cm^−1^ 3383, 2985, 1600, 1564, 1541, 1506; ^1^H-NMR (CDCl_3_): *δ* 2.49 (s, 6H, 2CH_3_), 6.68 (s, 1H, Ar Pyr), 7.86 (d, *J* = 8.7, 2H, Ar), 8.21 (d, *J* = 8.7, 2H, Ar), 8.70 (s, 1H, NH); ^13^C-NMR (CDCl_3_): *δ* 23.7, 113.3, 118.5, 125.4, 142.4, 144.9, 156.9, 168.1; ESI-HRMS [M + H]^+^ found = 245.10289, C_12_H_13_N_4_O_2_ requires = 245.10330.

#### *N*-(Biphenyl-4-yl)-4,6-dimethyl-2-pyrimidinamine (11)

Yield = 91%; light brown solid; mp 114–115.5 °C. It was obtained from 169 mg of 4-aminobiphenyl and purified by column chromatography starting from CH_2_Cl_2_–hexane (2 : 8), to CH_2_Cl_2_ as the eluent.

IR (KBr) *ν*_max_ cm^−1^ 3242, 2962, 1602, 1573, 1561, 1534; ^1^H-NMR (CDCl_3_): *δ* 2.41 (s, 6H, 2CH_3_), 6.50 (s, 1H, Ar Pyr), 7.31–7.36 (m, 1H, Ar), 7.43–7.48 (m, 2H, Ar), 7.58–7.64 (m, 4H, Ar, s, 1H, NH), 7.78–7.82 (m, 2H, Ar), ^13^C-NMR (CDCl_3_): *δ* 23.9, 111.7, 119.2, 126.7, 127.5, 128.8, 134.8, 139.4, 140.9, 159.6, 167.6; ESI-HRMS [M + H]^+^ found = 276.14892, C_18_H_18_N_3_ requires = 276.14952.

#### 4,6-Dimethyl-*N*-{4-[(*E*)-phenyldiazenyl]-phenyl}-2-pyrimidinamine (12)

Yield = 91%; red solid; mp 159.5–161 °C. It was obtained from 197 mg of 4-aminobenzene and purified by column chromatography using hexane–ethyl acetate (9 : 1) as the eluent.

IR (KBr) *ν*_max_ cm^−1^ 3278, 2960, 1604, 1585, 1562, 1535; ^1^H-NMR (CDCl_3_): *δ* 2.39 (s, 6H, 2CH_3_), 6.52 (s, 1H, Ar Pyr), 7.39–7.52 (m, 3H, Ar), 7.83–7.96 (m, 7H, Ar, NH); ^13^C-NMR (CDCl_3_): *δ* 23.9, 112.5, 118.5, 122.6, 124.2, 129.1, 130.3, 142.9, 147.5, 152.9, 159.1, 167.7; ESI-HRMS [M + H]^+^ found = 304.15492, C_18_H_18_N_5_ requires = 304.15567.

#### {3-[(4,6-Dimethylpyrimidine-2-yl)-amino]-phenyl}-methanol (13)

Yield = 97%; yellow solid; mp 106–108 °C. It was obtained from 123 mg of 3-aminobenzyl alcohol and purified by column chromatography starting from hexane to hexane–ethyl acetate (1 : 1) as the eluent.

IR (KBr) *ν*_max_ cm^−1^ 3271, 2939, 1618, 1593, 1556, 1548; ^1^H-NMR (CDCl_3_): *δ* 2.32 (s, 6H, 2CH_3_), 3.73 (bs, 1H, OH), 4.62 (s, 2H, CH_2_), 6.43 (s, 1H, Ar Pyr), 6.93–6.95 (m, 1H, Ar), 7.19–7.25 (m, 1H, Ar), 7.37 (s, 1H, NH), 7.49 (bs, 1H, Ar), 7.58–7.61 (m, 1H, Ar); ^13^C-NMR (CDCl_3_): *δ* 23.8, 64.8, 111.6, 117.5, 118.1, 120.7, 128.9, 139.9, 141.9, 159.4, 167.6; ESI-HRMS [M + H]^+^ found = 230.12849, C_13_H_16_N_3_O requires = 230.12879.

#### *N*-(3-Fluorophenyl)-4,6-dimethyl-2-pyrimidinamine (14)

Yield = 97%; white solid; mp 132–134 °C. It was obtained from 111 mg of 3-fluoroaniline and purified by column chromatography starting from CH_2_Cl_2_ to CH_2_Cl_2_–isopropanol (95 : 5) as the eluent.

IR (KBr) *ν*_max_ cm^−1^ 3419, 2922, 1602, 1587, 1566, 1539; ^1^H-NMR (CDCl_3_): *δ* 2.37 (s, 6H, 2CH_3_), 6.51 (s, 1H, Ar Pyr), 6.63–6.71 (m, 1H, Ar), 7.12–7.24 (m, 2H, Ar), 7.62 (s, 1H, NH), 7.78–7.83 (m, 1H, Ar); ^13^C-NMR (CDCl_3_): *δ* 23.9, 105.9 (d, *J* = 27.0), 108.5 (d, *J* = 22), 112.2, 114.1 (d, *J* = 2.5), 129.8 (d, *J* = 9.9), 141.7 (d, *J* = 5.8), 159.4, 163.3 (d, *J* = 242), 167.7; ESI-HRMS [M + H]^+^ found = 218.10851, C_12_H_13_FN_3_ requires = 218.10880.

#### 3-[(4,6-Dimethylpyrimidine-2-yl)-amino]-phenol (15)

Yield = 85%; brown solid; mp 137.5–139 °C. It was obtained from 109 mg of 3-aminophenol and purified by column chromatography starting from CH_2_Cl_2_–isopropanol (99 : 1) to CH_2_Cl_2_–isopropanol (8 : 2) as the eluent.

IR (KBr) *ν*_max_ cm^−1^ 3365, 2995, 1620, 1610, 1589, 1568, 1548; ^1^H-NMR (acetone d6): *δ* 2.30 (s, 6H, 2CH_3_), 6.45–6.48 (m, 1H, Ar), 6.54 (s, 1H, Ar Pyr), 7.05–7.11 (m, 1H, Ar), 7.26–7.30 (m, 1H, Ar), 7.66–7.68 (m, 1H, Ar), 8.37 (s, 1H, NH); ^13^C-NMR (acetone d6): *δ* 23.8, 106.6, 109.1, 110.8, 111.7, 129.9, 142.9, 158.5, 160.8, 168.1; ESI-HRMS [M + H]^+^ found = 216.11285, C_12_H_14_N_3_O requires = 216.11314.

#### 4,6-Dimethyl-*N*-(2-methylphenyl)-2-pyrimidinamine (16)

Yield = 90%; red solid; mp 111–112 °C. It was obtained from 107 mg of 4-toluidine and purified by column chromatography starting from hexane to hexane–ethyl acetate (8 : 2) as the eluent.

IR (KBr) *ν*_max_ cm^−1^ 3218, 2964, 1600, 1568, 1555; ^1^H-NMR (CDCl_3_): *δ* 2.32 (s, 3H, CH_3_), 2.36 (s, 6H, 2CH_3_), 6.48 (s, 1H, Ar Pyr), 6.87 (s, 1H, 1N), 6.96–7.01 (m, 1H, Ar), 7.17–7.25 (m, 2H, Ar), 8.17–8.19 (m, 1H, Ar); ^13^C-NMR (CDCl_3_): *δ* 18.3, 24.0, 111.7, 120.9, 122.9, 126.6, 127.7, 130.5, 137.9, 160.1, 167.7; ESI-HRMS [M + H]^+^ found = 214.13352, C_13_H_16_N_3_ requires = 214.13387.

#### 4,6-Dimethyl-*N*-(2-benzylamino)-2-pyrimidinamine (17)

Yield = 95%; white solid; mp 109–111 °C. It was obtained from 107 mg of benzylamine and purified by column chromatography starting from hexane to hexane–ethyl acetate (8 : 2) as the eluent.

IR (KBr) *ν*_max_ cm^−1^ 3254, 2964, 1600, 1583, 1570, 1559; ^1^H-NMR (CDCl_3_): *δ* 2.26 (s, 3H, CH_3_), 4.66 (d, *J* = 5.7, 2H), 5.85 (s, 1H), 6.29 (s, 1H, Ar Pyr), 7.22–7.36 (m, 5H, Ar); ^13^C-NMR (CDCl_3_): *δ* 24.0, 45.3, 109.9, 127.0, 127.5, 128.5, 139.7, 162.2, 167.5; ESI-HRMS [M + H]^+^ found = 214.13349, C_13_H_16_N_3_ requires = 214.13387.

### Procedure for the synthesis of compound (18)

#### *N*,*N*'-[Dithiobis(4,1-phenylene)]bis(4,6-dimethylpyrimidin-2-amine) (18)

Yield = 90%; yellow solid; mp 169.5–170.5 °C. It was obtained from 124 mg of 4,4′-dithiobis(benzenamine) and 142 mg of 2-chloro-4,6-dimethylpyrimidine and purified by column chromatography using hexane–ethyl acetate (3 : 2) as the eluent.

IR (KBr) *ν*_max_ cm^−1^ 3259, 2962, 1610, 1580, 1568, 1563, 1553; ^1^H-NMR (CDCl_3_): *δ* 2.35 (s, 6H, CH_3_), 6.48 (s, 1H, Ar Pyr), 7.42 (d, *J* = 8.6, 2H, Ar), 7.61 (d, *J* = 8.6, 2H, Ar), 7.76 (s, 1H, 1N); ^13^C-NMR (CDCl_3_): *δ* 23.9, 112.0, 119.2, 129.6, 131.2, 140.1, 159.3, 167.6; ESI-HRMS [M + H]^+^ found = 461.15649, C_24_H_25_N_6_S_2_ requires = 461.15766.

## Conflicts of interest

There are no conflict to declare.

## Supplementary Material

RA-010-D0RA00833H-s001
