# Supplementary material for: Microwave-assisted simple synthesis of 2-anilinopyrimidines by the reaction of 2-chloro-4,6-dimethylpyrimidine with aniline derivatives
Source: RSC Adv. 2020 Mar 25;10(21):12249–54. doi: 10.1039/d0ra00833h (PMC9050660; doi:10.1039/d0ra00833h)
Supplement: RA-010-D0RA00833H-s001 [file RA-010-D0RA00833H-s001.pdf]

Electronic Supplementary Material (ESI) for RSC Advances.
This journal is © The Royal Society of Chemistry 2020

Microwave-assisted simple synthesis of 2-anilinopyrimidines by the reaction of 2-chloro-4,6-dimethylpyrimidine with aniline derivatives

Cristina Campestre [a], György Keglevich [b], János Kóti [c], Luca Scotti [d], Carla Gasbarri [a] and Guido Angelini [a,*]

[a] Department of Pharmacy, University "G. d'Annunzio" of Chieti-Pescara, via dei Vestini, 66100 Chieti, Italy

[b] Department of Organic Chemistry and Technology, Budapest University of Technology and Economics, 1521 Budapest, Hungary

[c] Spectroscopic Research Division, Gedeon Richter Plc., 1475 Budapest, Hungary

[d] Department of Medical, Oral and Biotechnological Sciences, University "G. d'Annunzio" of Chieti-Pescara, via dei Vestini, 66100 Chieti, Italy

*Corresponding author (G. Angelini) guido.angelini@unich.it; Tel.: +39-0871-3554785

A. **Spectra of compound 11**
   Fig.1 ^{1}H-NMR spectrum of compound 11 in $CDCl_3$.
   Fig.2 ^{13}C-NMR spectrum of compound 11 in $CDCl_3$.
   Fig.3 HRMS of compound 11.

B. **Spectra of compound 12**
   Fig.4 ^{1}H-NMR spectrum of compound 12 in $CDCl_3$.
   Fig.5 ^{13}C-NMR spectrum of compound 12 in $CDCl_3$.
   Fig.6 HRMS of compound 12.

C. **Spectra of compound 18**
   Fig.7 ^{1}H-NMR spectrum of compound 18 in $CDCl_3$.
   Fig.8 ^{13}C-NMR spectrum of compound 18 in $CDCl_3$.
   Fig.9 HRMS of compound 18.

D. **Microwave conditions for the synthesis of compound 1**

A. Spectra of compound 11

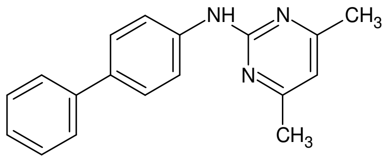

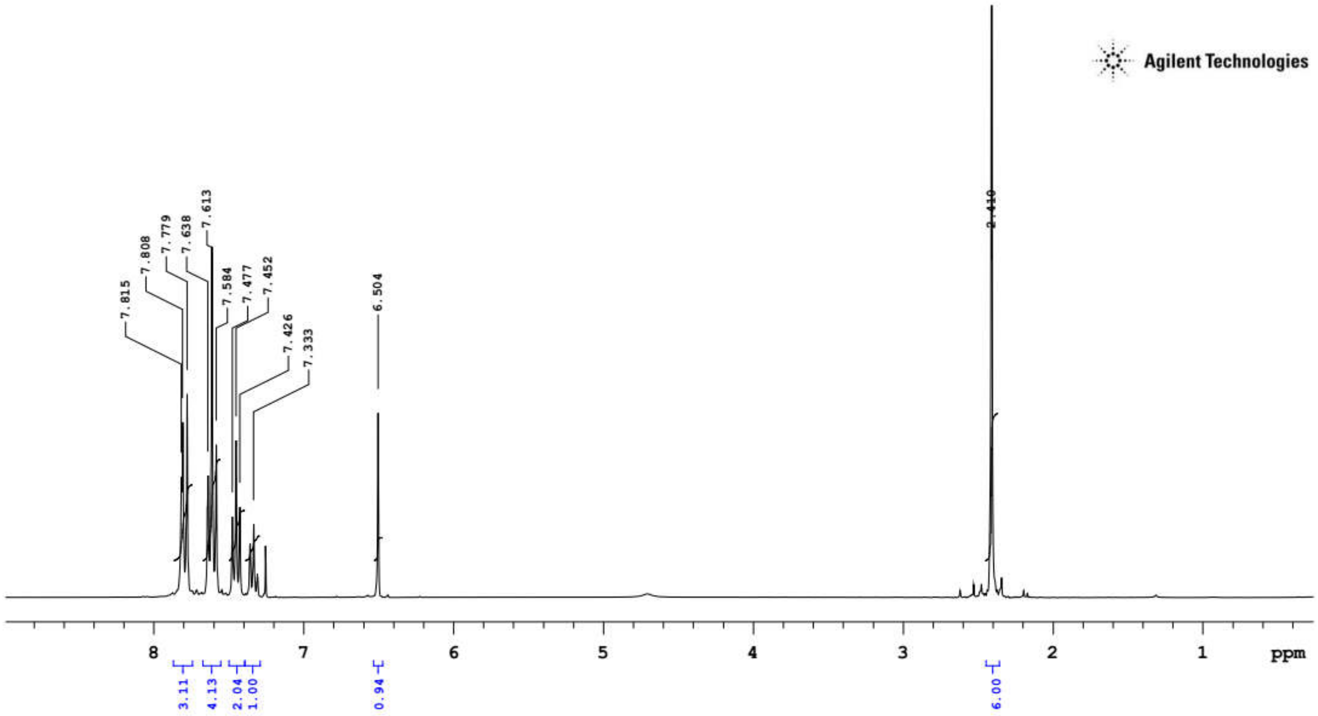

Fig.1 ^{1}H-NMR spectrum of compound 11 in $CDCl_3$.

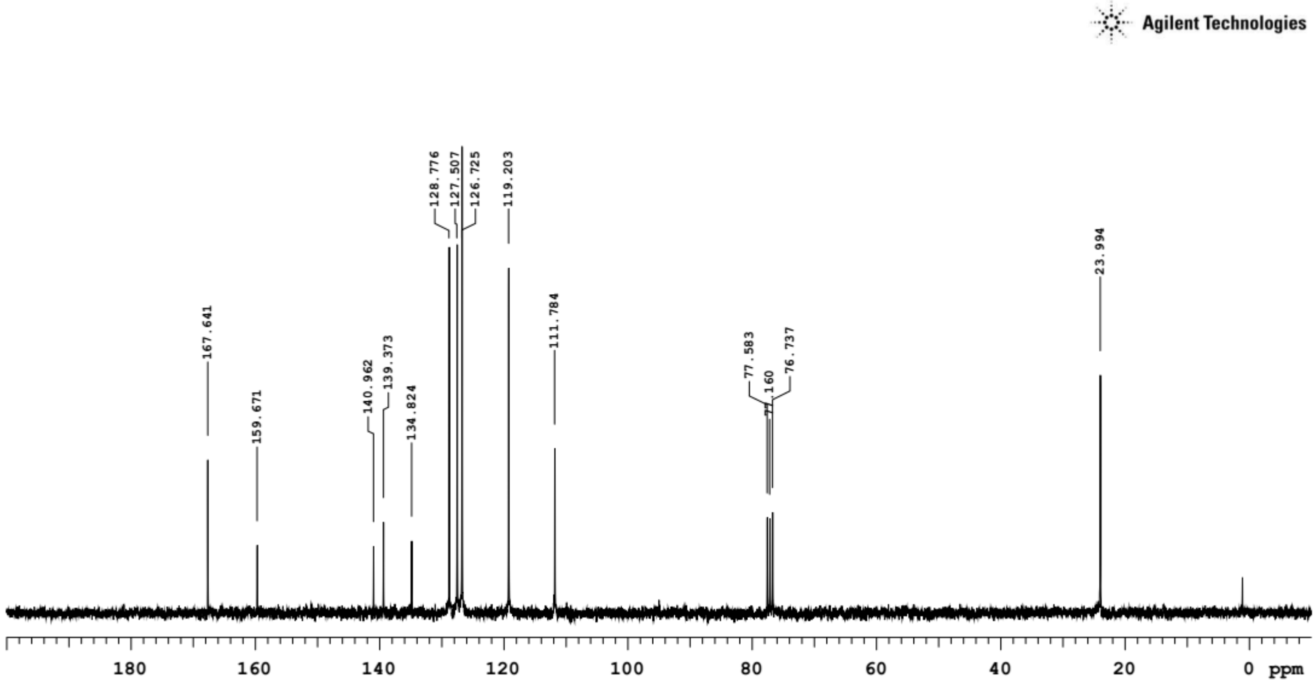

Fig.2 ^{13}C-NMR spectrum of compound 11 in $CDCl_3$.

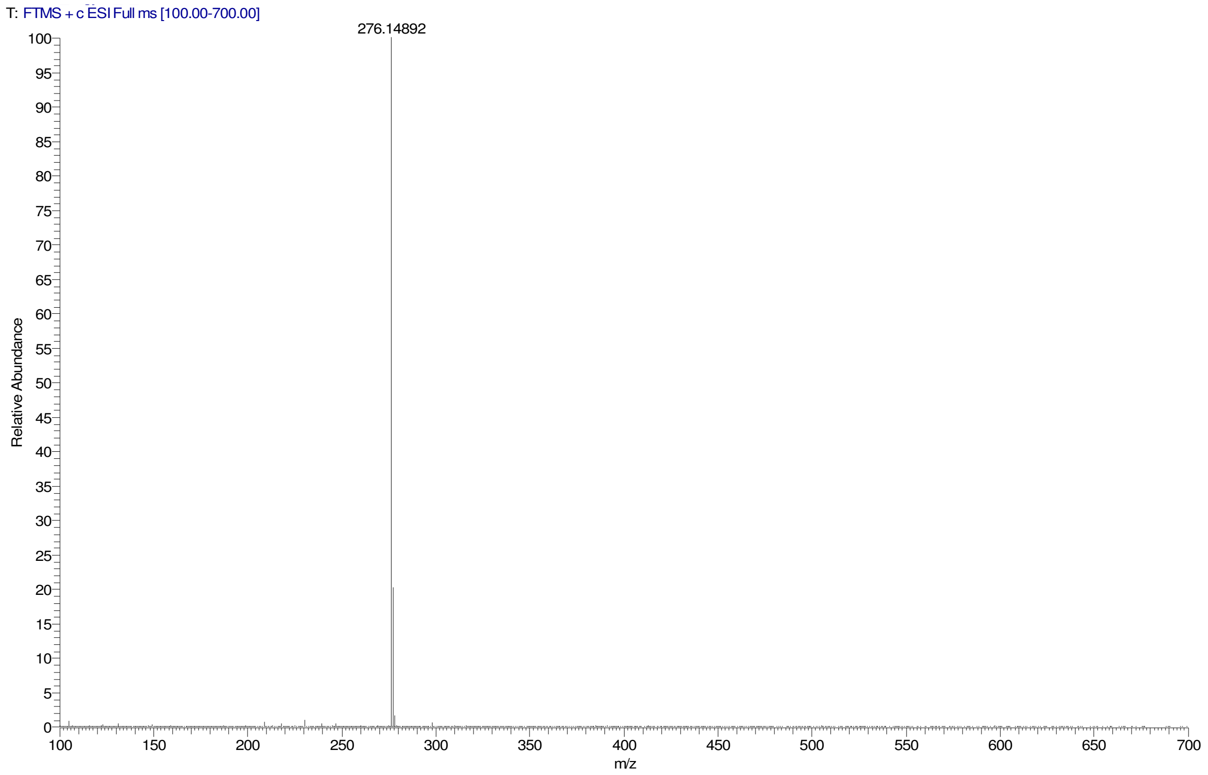

Fig.3 HRMS of compound 11.

B. Spectra of compound 12

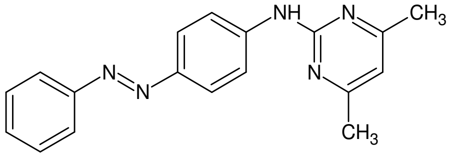

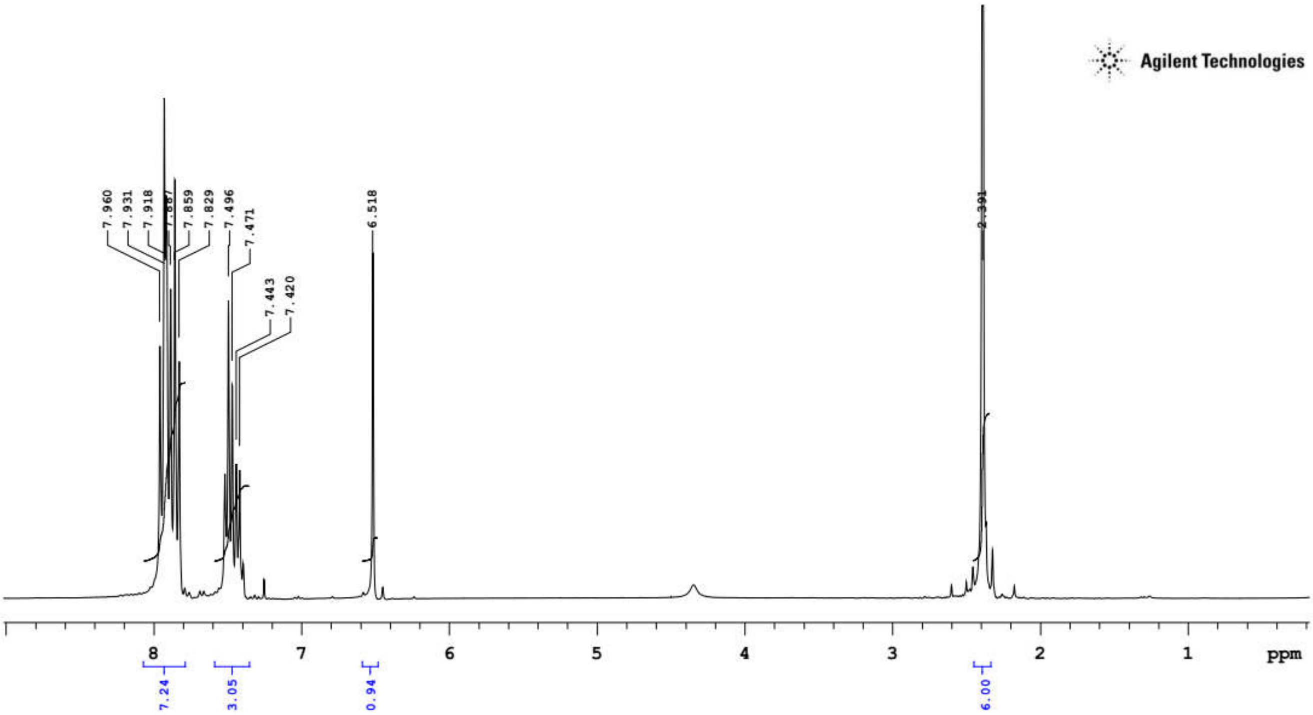

Fig.4 ^{1}H-NMR spectrum of compound 12 in $CDCl_3$.

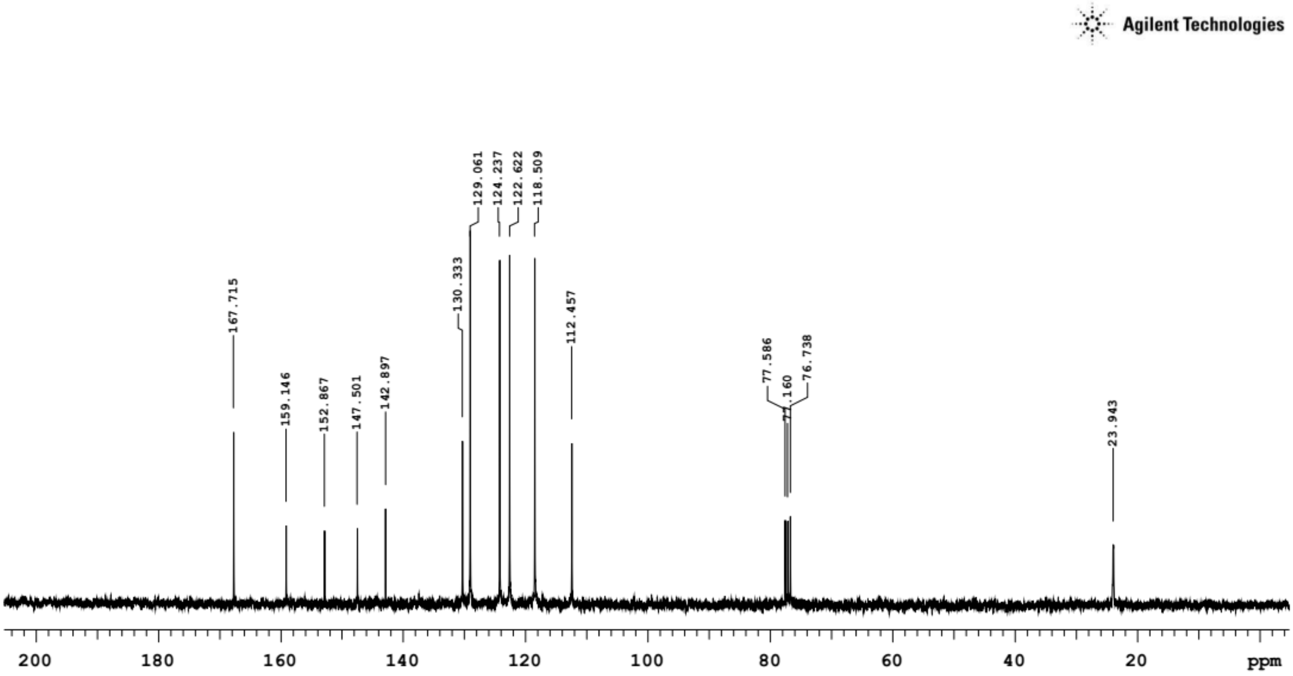

Fig.5 ^{13}C-NMR spectrum of compound 12 in $CDCl_3$.

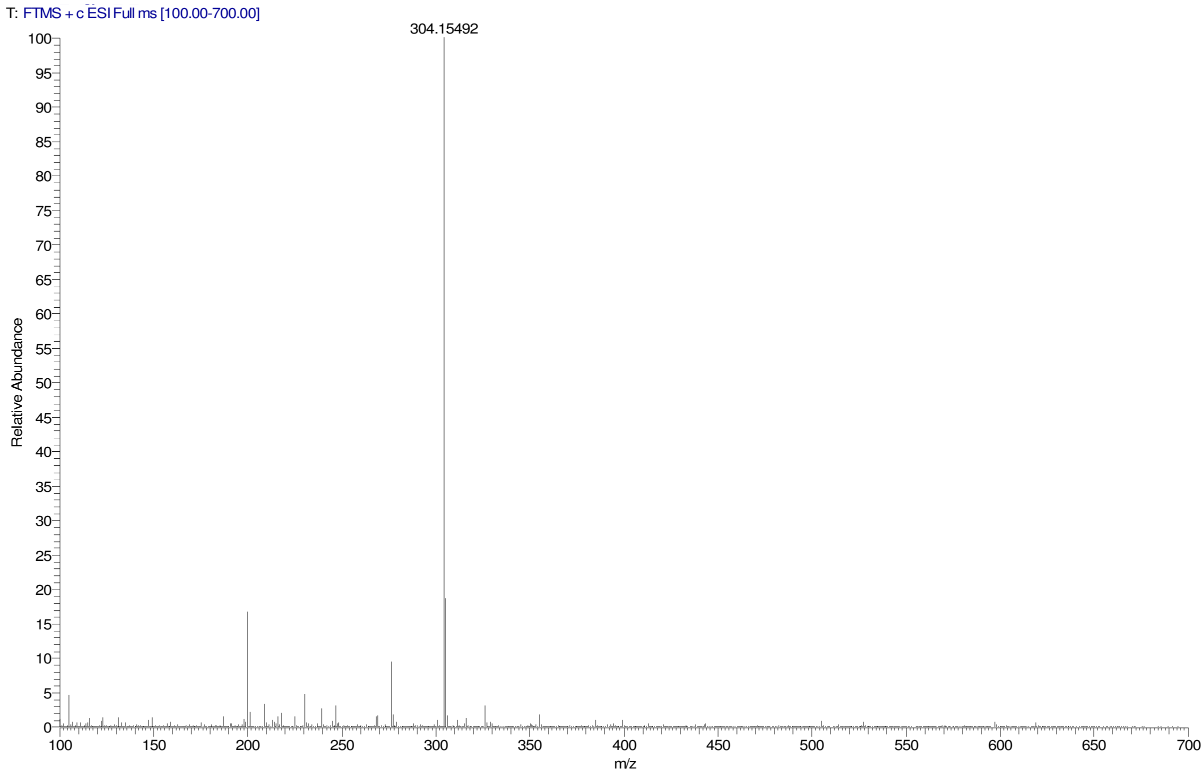

Fig.6 HRMS of compound 12.

C. Spectra of compound 18

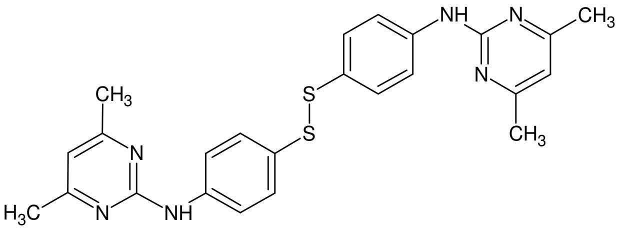

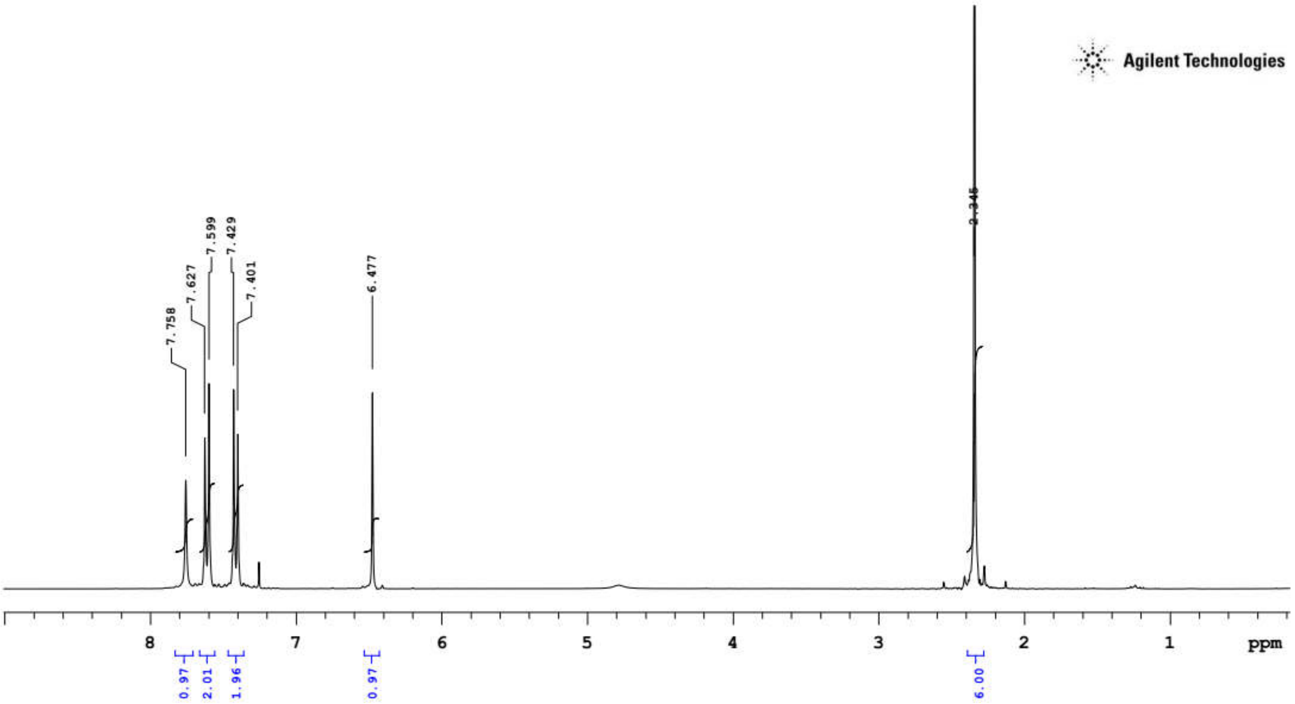

Fig.7 ^{1}H-NMR spectrum of compound 18 in $CDCl_3$.

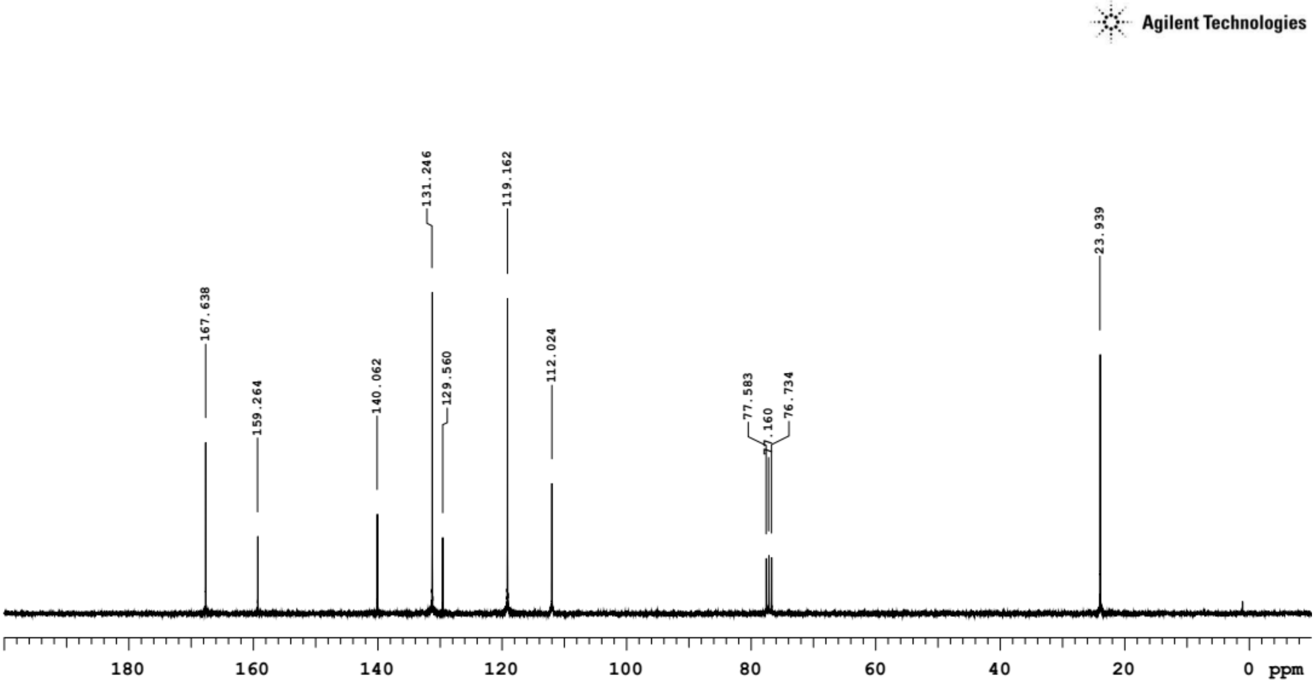

Fig.8 ^{13}C-NMR spectrum of compound 18 in $CDCl_3$.

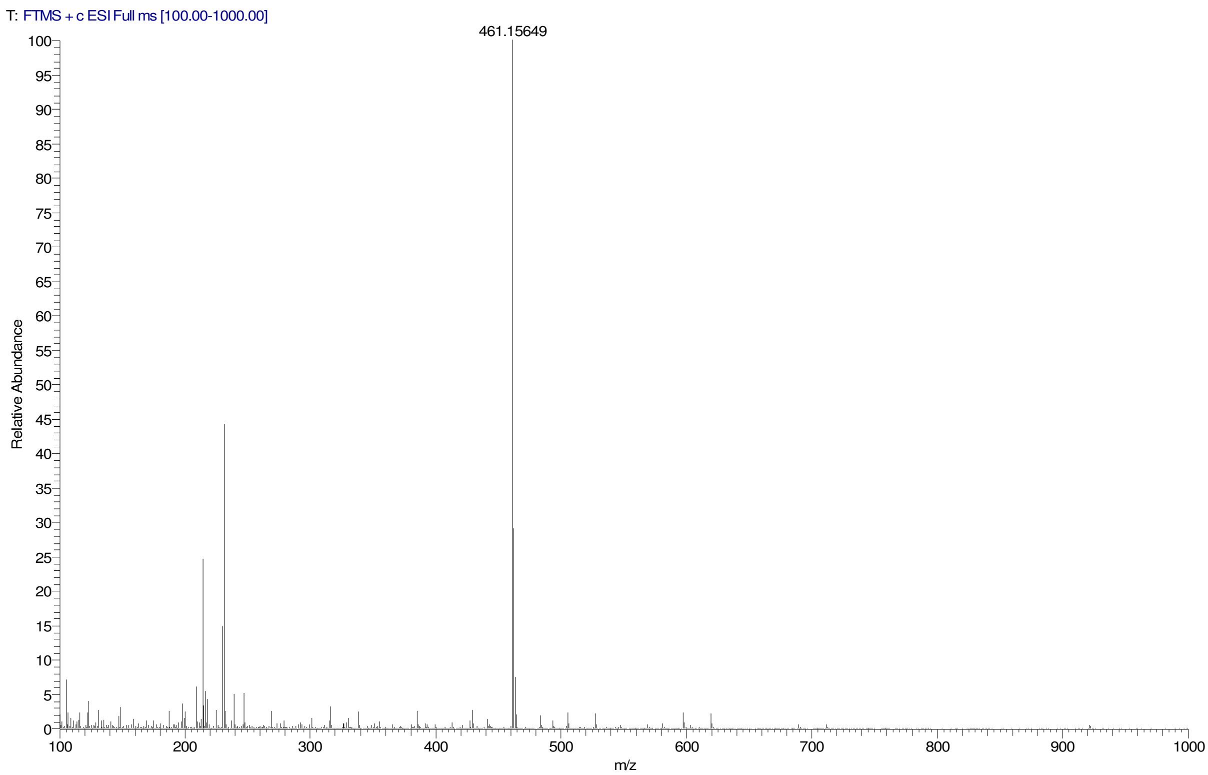

Fig.9 HRMS of compound 18.

D. Table S1. Microwave conditions for the synthesis of compound 1

Time / min	T / °C	Yield / %
5	130	17
10	130	49
15	130	71
30	130	80
20	140	90
10	150	88
10	160	90
